# Nutritive Value of Tomato Pomace for Ruminants and Its Influence on In Vitro Methane Production

**DOI:** 10.3390/ani9060343

**Published:** 2019-06-12

**Authors:** Carlos N. Marcos, Trinidad de Evan, Eduarda Molina-Alcaide, M. D. Carro

**Affiliations:** 1 Departamento de Producción Agraria, Escuela Técnica Superior de Ingeniería Agronómica, Agroalimentaria y de Biosistemas, Universidad Politécnica de Madrid, Ciudad Universitaria, 28040 Madrid, Spain; navarro-88@hotmail.com (C.N.M.); tdeevan@ucm.es (T.d.E.); 2 Estación Experimental del Zaidin (Consejo Superior de Investigaciones Científicas), Profesor Albareda 1, 18008 Granada, Spain; molina@eez.csic.es

**Keywords:** tomato pomace, in vitro rumen fermentation, methane, rumen degradability, intestinal digestibility

## Abstract

**Simple Summary:**

Reutilization of agroindustrial by-products in animal feeding could contribute to the reduction of environmental problems associated with their accumulation, but it is necessary to assess their nutritive value. Tomato pomace (TP) is a by-product of the tomato industry that could be used in ruminant feeding, but data on its nutritive value are limited. The aim of this work was to analyze the chemical composition, in vitro rumen fermentation, and intestinal digestibility of 12 TP samples obtained from two processing plants at different times during the tomato campaign and to assess the in vitro fermentation of diets including increased TP amounts. The chemical composition of TP showed little variability. Samples of TP had high fiber, protein, and fat content and were rapidly fermented in the rumen, but the in vitro intestinal digestibility of the protein was low. The in vitro results provide useful information for including TP in ruminant diets, indicating that amounts of TP up to 180 g/kg could be included in a diet for fattening ruminants without negatively affecting rumen fermentation, but these results should be confirmed in vivo.

**Abstract:**

The objective of this study was to determine the variability in nutritive value for ruminants of tomato pomace (TP) samples and analyze its effect on in vitro fermentation when it was included in a high-concentrate diet. Twelve TP samples were obtained from two processing plants at weekly intervals and analyzed for chemical composition, in vitro rumen fermentation, and intestinal digestibility. The chemical composition of TP did not differ between processing plants and only slight variations were observed among sampling times. Tomato pomace had a low dry matter content (<300 g/kg), a high content of neutral detergent fiber, crude protein, and ether extract (572, 160, and 82.7 g/kg dry matter on average, respectively), and was rapidly fermented in the rumen. Protein degradability at 16 h in situ incubation was 510 g/kg and in vitro intestinal digestibility of protein was low (430–475 g/kg). Replacing soybean meal and barley straw by dried TP increased the in vitro fermentation rate and the production of volatile fatty acids and reduced NH_3_-N concentrations without affecting CH_4_. In summary, TP samples showed little variability in nutritive value over sampling time and TP of up to 180 g/kg could be included in high-concentrate diets without negatively affecting rumen fermentation.

## 1. Introduction

The world production of processing tomato has increased up to 37.8 × 10^6^ tons in 2017 according to estimates of the World Processing Tomato Council [1]. In the European Union, Italy and Spain are the greatest tomato producers, accounting for 4.7 × 10^6^ and 2.8 × 10^6^ tons of tomatoes for processing in 2017, respectively. Tomato pomace (TP) is a by-product of the tomato industry that consists mainly of peels, seeds, and small quantities of pulp that remain after the production of juice, paste, sauce, and other processed products, representing between 5% and 10% of the original fruits [2]. The high water content of TP (usually greater than 75%) due to the water added in the last step of the industrial process is the most important limitation for its further utilization in animal feeding, as fresh TP spoils very quickly [3]; therefore, the use of fresh TP for animal feeding is generally restricted to farms closely located to the processing plants [4], also limited because high-moisture feeds reduce feed intake in ruminants. Tomato pomace can be preserved either by drying or by ensiling with cereal straw [4], but these procedures increase their price. The use of TP in animal feeding would contribute to the reduction of environmental problems caused by their accumulation, mainly related to bad odor and pests breeding in accumulations of spoiled TP [5]. In addition, TP can improve the quality of animal products by increasing the content of fatty acids beneficial to human health [6,7]. However, the nutritive value of TP depends on many factors, such as cultivars, season, or processing, among others [8], but current knowledge on its variability is limited.

Previous studies have shown that the inclusion of tomato fruit wastes in the diet of goats reduced CH_4_ emissions [9,10] and this antimethanogenic effect has also been observed in vitro [11]. The antimethanogenic mechanisms of tomato fruit wastes are still unknown [10], but the presence of bioactive substances affecting the rumen methanogenic archaea cannot be ignored. However, to our best knowledge, the possible influence of TP on rumen CH_4_ production has not yet been tested. Therefore, the first objective of this study was to analyze the variability in the nutritive value of TP samples collected from tow processing plants during the tomato campaign. The second objective was to assess the effects of including increasing doses of TP in a diet for fattening lambs on in vitro fermentation and CH_4_ production.

## 2. Materials and Methods

### 2.1. Tomato Pomace Samples

Twelve samples of TP were obtained from two of the biggest Spanish tomato processing plants (Tomates del Guadiana Soc. Coop., Badajoz, Spain, and PRONAT, Badajoz, Spain) during the 2016 tomato harvesting campaign. The tomato campaign in Spain is usually concentrated in August and September. Samples (4–5 kg) were obtained weekly at each plant on the 12th, 19th, and 26th August and the 2nd, 9th, and 16th of September, frozen (−20 °C) and lyophilized. In all cases, samples were the final tomato-processing disposal. All samples were ground at 2 mm, and subsamples were ground at 1 mm before chemical analyses and in vitro incubations.

### 2.2. Animals and Feeding

Four adult rumen-fistulated Lacaune sheep (63.5 ± 2.05 kg body weight (BW)) were individually housed and had free access to fresh water over the trial. Animals were fed twice daily (8:00 and 18:00) a 2:1 grass hay:concentrate diet at a rate of 45 g dry matter (DM)/kg BW^0.75^. The diet contained 114, 365, and 160 g of crude protein (CP), neutral detergent fiber (NDF), and acid detergent fiber (ADF) per kg DM, respectively, and was formulated to meet maintenance requirements of the experimental animals [12]. Sheep were managed in accordance with the Spanish guidelines for experimental animal protection (Royal Decree 53/2013 of 1st February on the protection of animals used for experimentation or other scientific purposes) and all experimental procedures were approved by the Institutional Animal Care and Use Committee of the Comunidad Autónoma de Madrid (PROEX 035/17).

### 2.3. Experimental Design

The study comprises two experiments. The in vitro rumen fermentation and the intestinal digestibility of protein (IDCP) of the TP samples were measured in Experiment 1, whereas Experiment 2 was designed to analyze the effects of including dried TP in the diet on in vitro rumen fermentation, with special emphasis on CH_4_ production.

#### 2.3.1. Experiment 1: In Vitro Fermentation of TP Samples

Two in vitro trials were conducted on different days using the same methodology and in each of them there were four replicates per TP sample by using the ruminal fluid from each sheep as inoculum.

The first trial was carried out to determine the gas production kinetics of samples and the second one to assess the main fermentation parameters and CH_4_ production. Ruminal contents of each sheep were obtained before the morning feeding, strained through four layers of cheesecloth into previously warmed thermal flasks, and immediately transported to the laboratory. The fluid of each sheep was independently mixed with pre-warmed (39 °C) culture medium (Goering and Van Soest [13]; without trypticase) in a proportion 1:4 under CO_2_ flushing. The medium of Goering and Van Soest [13] was modified by replacing the (NH_4_)HCO_3_ with NaHCO_3_ and excluding the trypticase to avoid N supply. Samples (200 mg of DM) of each feed were accurately weighed into 60-mL vials. Vials were filled up with 20 mL of the rumen fluid—culture medium mixture using a Watson–Marlow 520UIP31 peristaltic pump (Watson–Marlow Fluid Technology Group, Cornwall, UK), sealed with rubber stoppers, and incubated at 39 °C for 120 h. Gas production was measured at 3, 6, 9, 12, 15, 22, 26, 31, 36, 48, 58, 72, 96, and 120 h using a pressure transducer (Delta Ohm DTP704-2BGI, Herter Instruments SL, Barcelona, Spain) and a plastic syringe and the gas produced at each sampling time was released to prevent gas accumulation. Two blanks (vials without sample) per inoculum were also incubated in this trial to correct gas production values for endogenous production. The potential in vitro DM degradability (PDMD) was determined by weighing 300 mg of each feed (3 bags per feed) into polyester bags of 30 µm pore size (Ankom Corp #57, Ankom Technology Corp., Fairport, NY, USA) and incubating the bags in a 1:4 mixture of ruminal fluid and the incubation medium of Goering and Van Soest [13] in an Ankom Daisy II incubator at 39 °C under continuous rotation for 120 h. A mixture of equal parts of the ruminal fluid from each sheep was used as inoculum. After 120 h, bags were washed with cold water, dried at 60 °C for 48 h, and weighed to calculate PDMD.

In the second in vitro trial, vials were incubated at 39 °C for 24 h as described before and samples to analyze the main fermentation parameters were taken. Gas production was measured and a gas sample (10 mL) was stored in an evacuated tube (Terumo Europe N.V., Leuven, Belgium) for analysis of CH_4_ concentration. Vials were then uncapped, their content was homogenized, the pH was immediately measured (Crison Basic 20 pH-meter, Crisson Instruments, Barcelona, Spain), and 3 mL of the vials’ contents were mixed with 3 mL of 0.5 M HCl. Samples were frozen (−20 °C) until volatile fatty acid (VFA) and NH_3_-N analyses.

#### 2.3.2. Experiment 1: In Vitro Intestinal Digestibility of Tomato Pomace Samples

The determination of the IDCP of TP samples followed the three-step procedure described by Gargallo et al. [14] and was performed as detailed by Moshen et al. [15]. Briefly, 3 g of each TP sample (2 mm size) was weighed into nylon bags (7 × 14 cm; 46 μm pore size) and incubated in the rumen of each sheep immediately before the afternoon feeding on 2 different days. After 16 h of in situ incubation, bags were washed, frozen (−20 °C, 48 h) to facilitate the detachment of particle-associated bacteria, thawed, washed 3 times with cold water (5 min each) in a turbine washing machine, frozen again (−20 °C), lyophilized, and weighed. Finally, bag residues were analyzed for N content to calculate the in situ degradability of CP. Bag residues were pooled by TP sample and sheep and 0.5 g of each were weighed in duplicate into nylon bags (5 × 5 cm; 46 μm pore size). Bags were incubated into Daisy II incubation bottles (ANKOM Tech. Co., Fairport, NY, USA) containing 2 liters of a 0.1 N HCl solution (pH 1.9; 39 °C) with 1 g per l of pepsin (P-7000, Sigma, St. Louis, MO, USA) under constant rotation at 39 °C for 1 h. Bags were then rinsed with tap water and incubated in a pancreatin solution (0.5 M KH_2_PO_4_ buffer, pH 7.75, 50 ppm of thymol and 3 g per l of pancreatin; P-7545, Sigma, St. Louis, MO, USA) at 39 °C for 24 h under constant rotation. Finally, bags were rinsed with tap water, dried at 40 °C for 72 h, and weighed. Residues of the in vitro incubation were pooled by sheep and TP sample and analyzed for N concentration to calculate IDCP values.

#### 2.3.3. Experiment 2: In Vitro Fermentation of Diets Containing TP

The objective of this experiment was to analyze the effects of including increasing amounts of dried TP in a high-concentrate diet for fattening lambs (control diet) on in vitro rumen fermentation. Three additional diets were formulated by replacing different amounts of barley straw, soybean meal, and wheat bran in the control diet with 60, 120, or 180 g per kg (fresh matter basis) of a dried TP sample. A composited sample of the TP samples tested in Experiment 1 was used and it contained 967, 163, 572, 446, and 95.2 g of organic matter (OM), CP, NDF, ADF and ether extract (EE) per kg DM, respectively. All diets were formulated to have similar CP and NDF content to the control diet and therefore TP replaced fibrous (barley straw and wheat bran) and protein (soybean meal) conventional ingredients. The ingredients and chemical compositions of the four experimental diets are given in Table 1.

All diets were incubated in vitro for 24 h following the methodology described in Experiment 1, with the exception that the amount of sample was increased to get enough gas for the analysis of CH_4_ concentration; therefore, 400 mg of each diet was fermented with 40 mL of the ruminal fluid and culture medium mixture in 120-mL vials. After 8 h of incubation, gas production was measured and the gas was sampled for CH_4_ analyses as described in Experiment 1. Immediately, 1 mL of each vial content was taken using an insulin syringe, mixed with 1 mL of 0.5 M HCl, and stored at −20 °C for VFA and NH_3_-N analyses. After 24 h, the gas produced and the pH of the vials’ contents were measured and samples for CH_4_, VFA and NH_3_-N analyses were taken. Four replicates per diet were obtained by using the ruminal fluid from each sheep independently as inoculum.

### 2.4. Chemical Analyses

Chemical composition of TP samples and feed ingredients of experimental diets were analyzed using the Association of Official Analytical Chemists [16] procedures for DM (ID 934.01), ash (ID 048.13), and EE (ID 945.16). Concentrations of NDF, ADF, and lignin were determined following the procedures of Van Soest et al. [17] and Robertson and Van Soest [18], respectively, using an ANKOM220 Fiber Analyzer unit (ANKOM Technology Corporation, Fairport, NY, USA). Sodium sulphite and α-amylase were used in the sequential analysis of NDF, ADF, and lignin and ash-free values are reported. Nitrogen was measured by the Dumas combustion method employing a Leco FP258 N Analyzer (Leco Corporation, St. Joseph, MI, USA) and the amount of acid detergent insoluble N (ADIN) was determined by analyzing the N content in the residue obtained after the treatment of the sample with acid detergent solution. Total sugars and total soluble polyphenols (TSP) were analyzed by colorimetric methods following the anthrone method [19] and the Folin–Ciocalteu assay [20], respectively, using an Epoch spectrophotometer (BioTek Instruents Inc., Winooski, VT, USA). Concentrations of NH_3_-N were determined by the phenol-hypochlorite method [21] and those of VFA and CH_4_ by gas chromatography as described by García-Martínez et al. [22] and Martínez et al. [23], respectively. All analyses were performed in duplicate.

### 2.5. Calculations and Statistical Analyses

Data of gas production were fitted with time using the exponential model Gas = PGP × (1 − e^(−c × (t − lag))^), where PGP is the asymptotic gas production, c is the fractional rate of gas production, lag is the time before starting gas production, and t is the time of gas measurement. Gas production parameters were estimated using the NLIN procedure of SAS [24] by an iterative least squares procedure. The average gas production rate (AGPR) was calculated as AGPR = PGP × c ÷ [2 × (ln2 + c × lag)] and it was defined as the rate between the incubation start and the time at which half PGP is reached. The DM effective degradability (DMED) was estimated as DMED = [(PDMD × c) ÷ (c + Kp)] × e^(−c × lag)^, using a rumen particulate outflow (Kp) of 0.042 per h. Finally, the amount of fermented OM (FOM) was calculated from acetate, propionate, and butyrate production in each vial, as described by Demeyer [25].

As there were no differences (*p* ≥ 0.191) in the chemical composition of the TP samples between the 2 processing plants, chemical composition data were analyzed using the PROC GLM of SAS (SAS Institute, Cary, NC, USA) [24] with sampling time as the main effect. Data from Experiment 1 were analyzed as a mixed model with repeated measurements using the PROC MIXED of SAS, in which the effect of sampling time (1 to 6 weeks) was considered fixed and that of the inoculum was considered random. Data from Experiment 2 were analyzed as a mixed model, with the effect of the diet being fixed and that of the inoculum being random. Non-orthogonal polynomial contrasts were used to test for linear and quadratic effects of TP inclusion. Significance was declared at *p* < 0.05, whereas *p* < 0.10 values were considered as a trend. When a significant effect of sampling time was detected, means were compared by Tukey’s test. Relationships between chemical composition of the TP samples and either gas production or fermentation parameters were assessed by linear regression using the PROC CORR of SAS [24].

## 3. Results

### 3.1. Chemical Composition of TP Samples, In Vitro Fermentation, and Intestinal Digestibility of Crude Protein

Chemical composition of TP (Table 2) was relatively unchanged over the sampling period and only lignin and EE contents varied significantly (*p* ≤ 0.017) with time; in general, lignin increased with advancing time, whereas EE decreased. Both NDF and ADF contents tended (*p* = 0.096 and 0.063, respectively) to be greater in the last weeks of sampling compared with the first sampling week. Dry matter, total sugars, and TSP were the most variable fractions, with coefficients of variation (CV) of 21.9%, 20.1%, and 25.1%, respectively (results not shown), whereas the rest of the analyzed fractions showed a CV lower than 10%, except ADIN and EE (15.4% and 18.1%, respectively).

As shown in Table 3, there were differences (*p* ≤ 0.043) among TP samples taken at different times in all gas production and in vitro fermentation parameters, except for the fractional gas production rate, lag, NH_3_ concentrations, and CH_4_ production, although a trend (*p* ≤ 0.095) was detected for lag and CH_4_ production. Fermentation data indicated that TP samples taken at the end of the campaign were less degradable than the rest of samples.

Values of 16-h in situ degradability of DM ranged from 488 to 540 g/kg, and decreased (*p* = 0.005) as sampling time advanced (Table 4). In contrast, 16 h in situ degradability of CP, IDCP, and total CP digestibility values did not change (*p* ≥ 0.190) over the sampling period.

Table 5 shows the correlation coefficients between chemical composition and gas production parameters (PGP and AGPR), DMED, some fermentation parameters, in situ rumen degradability of DM and CP, and IDCP of TP samples. Organic matter was the chemical fraction more closely correlated (r ≥ 0.860; *p* < 0.001) with PGP, AGPR, and total VFA production; ADF was the only fraction significantly correlated (*p* = 0.030) with CH_4_ production. In situ DM degradability showed negative correlations (r ≥ −0.699; *p* ≤ 0.011) with NDF, ADF, and lignin, whereas in situ CP degradability was negatively correlated with ADF (r = −0.724; *p* = 0.008) and positively with CP content (r = 0.796; *p* = 0.002). The IDCP was negatively affected by TSP content (r = −0.744; *p* = 0.006), and total CP digestibility was positively correlated with CP content (r = 0.790; *p* = 0.002).

### 3.2. In Vitro Fermentation of Diets Containing TP

There were no differences (*p* ≤ 0.175) among diets (Table 6) in PGP, lag, AGPR, and DMED values, but the fractional rate of gas production was linearly (*p* = 0.016) and quadratically (*p* = 0.015) augmented with increasing TP amounts in the diet. Total VFA production at 8 h of incubation was not affected (*p* ≥ 0.161) by the inclusion of TP, but after 24 h of incubation a linear increase (*p* = 0.029) in total VFA production was observed. At 8 h of incubation, the VFA profile was modified by TP inclusion and there was an increase in molar proportion of acetate (quadratic; *p* = 0.038) and propionate (linear; *p* = 0.007) and a linear decrease in the proportion of butyrate (*p* = 0.009) and minor VFA (calculated as the sum of isobutyrate, isovalerate, and valerate; *p* = 0.002) as TP increased. However, after 24 h of incubation, only a linear increase (*p* = 0.002) in propionate proportion and a linear decrease (*p* = 0.002) in the proportion of minor VFA were observed. As a consequence, acetate/propionate ratios decreased linearly at both incubation times (*p* = 0.039 and 0.007 at 8 and 24 h, respectively). Concentrations of NH_3_-N decreased linearly at 8 h (*p* = 0.049) with increasing amounts of TP in the diet and a trend (*p* = 0.066) was detected after 24 h of incubation. Including TP in the diet did not affect CH_4_ production at any time (*p* ≥ 0.194), but a linear reduction (*p* = 0.047) of CH_4_/VFA ratio was observed at 8 h of incubation; however, this effect disappeared after 24 h of incubation.

## 4. Discussion

### 4.1. Chemical Composition, In Vitro Fermentation, and In Vitro Intestinal Digestibility of TP Samples

Chemical composition of TP was within the range of values previously reported [4,26,27], although other studies [2,28] reported lower lignin and greater EE values. Tomato pomace is a fibrous by-product with a relatively high content of CP and EE. As reported by others [8,29,30], most of fiber and CP comes from the peel fraction of TP, whereas seeds contain most of ash and EE; therefore the relative proportions of peels and seeds in TP can help to explain the observed differences in lignin and EE due to sampling time in the present study, as well as variations in chemical composition among studies [31]. In addition, chemical composition of tomato fruits has been reported to be highly variable, depending on factors such as cultivars, crop conditions, origin, etc. [8], which also contributes to variability in TP composition. Additional factors, such as tomato industrial processing characteristics, can increase variability. However, in the present study, there were no differences in any chemical fraction between the TP samples from the two processing plants, which might be due to a similar processing type in both of them. Finally, the application of high temperatures during tomato processing may result in the formation of indigestible compounds via the Maillard reactions between sugar aldehyde groups and free amino groups [32] that resulted in high ADIN values (16%–26%) reported in some studies [2,33,34]. However, ADIN concentrations in our study were relatively low, ranging from 6.6% to 8.8% of total N. Differences in the ADIN content could be attributed to variability in tomato fruits or processing [8], but also to different processing temperatures.

Despite the relatively low variability in TP chemical composition observed in our study, there were some differences between sampling times in PGP, AGPR, and DEMD, with samples taken at week 6 having lower values than those taken at week 2. Abbeddou et al. [26] pointed out that CP and non-structural carbohydrates of TP are easy and rapidly degraded, whereas NDF has low degradability. The low lag values (≤2.63 h) agree with this observation. These results indicate that TP was rapidly fermented in vitro.

Values of total VFA production agree well with gas production parameters, as TP samples taken at week 6 had lower values than the rest of the samples, except those taken at week 1. Despite the fact that there were significant effects of sampling period on molar proportions of all individual VFA, changes were of minor importance and the VFA profile remained relatively constant over the sampling period.

The main fatty acids present in TP are oleic and linoleic acids [35] and the unsaturated fatty acids which are produced by the hydrolysis of triglycerides are toxic to the fibrolytic bacteria [36]. However, the lack of correlation (*p* ≥ 0.339) between EE content and any gas production or in vitro fermentation parameter indicates that EE had no negative effect on TP fermentation, despite the fact that its concentrations were greater than the maximal level of 60 g of fat per kg of DM recommended in the diet of ruminants to avoid reductions in fiber digestibility [37]. There were some differences between samples in CH_4_ production, but these differences disappeared when the CH_4_/total VFA ratio was calculated.

Although OM content showed low variability among TP samples, it was the chemical fraction more closely related to gas production parameters and VFA and CH_4_ production, with the correlation being negative; this might be explained because of the close relationship between OM and the lignin/NDF ratio (r = 0.830; *p* < 0.001), as lignification is one of the main factors limiting NDF degradability [38]. In fact, the lignin/NDF ratio was negatively correlated with PGP, DMED, and total VFA, indicating that NDF lignification was a major factor involved in the differences observed in fermentation parameters.

Values of 16 h in situ rumen DM degradability were similar to those reported by Abbeddou et al. [26] and Gasa et al. [28] after 16 and 24 h of ruminal incubation, respectively (476 and 471 g/kg), but greater than the 389 g/kg reported by Fondevila et al. [2] after 24 h of incubation in the rumen. Values of in situ degradability of CP were greater than the 406 g/kg reported by Fondevila et al. [2] for 24 h of incubation, but lower than the 649 g/kg reported by Abbeddou et al. [26] for 16 h of incubation. Differences among studies are probably due to variability in TP chemical composition, but may be also related to the performance of the in situ procedure which is influenced by many factors, such as animals, diet, incubation procedure, etc. [39]. In situ degradability of both DM and CP was negatively correlated with ADF content, reflecting the low degradability of this fraction, which is the main constituent of fiber in TP; this might also restrict the access of microorganisms to protein.

The values of IDCP revealed that only nearly half of the CP undegraded in the rumen can be digested in the intestine, and total CP digestibility reached 737 g/kg as average. These values agree with previous observations reporting lower protein digestibility in TP compared with protein concentrates such as soybean meal [2,40]. The positive correlation of IDCP with ADF fraction is in accordance with the observation that intestinal digestibility of CP increases as rumen degradability of CP decreases [41]; this would explain the fact that ADF was negatively correlated with in situ CP degradability and positively with IDCP. The negative correlation between IDCP and TSP content may indicate that TSP formed less-digestible complexes with dietary proteins.

### 4.2. In Vitro Fermentation of Diets Containing TP

The increased fractional rates of gas production indicate that diets including TP were fermented more rapidly than the control diet, which is in agreement with the increased VFA productions observed at 24 h of incubation, although only TP18 diet showed significant differences with the control diet. These results are consistent with the lower amounts of barley straw in the TP-diets than in the control diet, as barley straw is slowly degraded in the rumen and greater VFA productions and FOM were observed for the TP-diets after 24 h of incubation. Including TP in the diet also changed the VFA profile. Our results are in agreement with those of Soto et al. [42], who observed that in vitro molar proportion of acetic acid increased and butyric and minor VFA proportions decreased when tomato fruit wastes were included in the diet. Similarly, Arco-Perez et al. [7] reported an increase in propionate proportions and a decrease in the acetate/propionate ratio in the rumen of goats receiving tomato silage compared with those fed a control diet.

The reduced NH_3_-N concentrations observed with increasing TP levels could be due to the lower degradation of TP protein, as NH_3_-N is one of the main final products of protein degradation in the rumen. The decreased proportions of minor VFA as the level of TP in the diet increased are consistent with this hypothesis, but NH_3_-N concentrations were adequate for rumen microorganism growth in all diets [43]. Tomato pomace replaced increased amounts of soybean meal in the experimental diets and rumen degradability of TP has been reported to be lower than that of soybean meal [2], which would indicate greater bypass protein in TP than in soybean meal. According to Drouliscos [44], the essential amino acids profile in TP protein is similar to that in soybean meal, but the low values of IDCP observed in our study indicate that about half of the protein reaching the small intestine may not be digested, whereas intestinal digestibility of soybean meal is greater than 90% [43]. In agreement with these results, Fondevila et al. [2] and Yuangklang et al. [40] reported a significant decrease in CP digestibility when replacing soybean meal by TP in the diet of lambs and beef cattle, respectively.

Although some studies have shown that the inclusion of tomato fruit wastes in the diet reduced CH_4_ emission both in goats [7,9,10] and in in vitro fermentations [11], no changes in CH_4_ production were observed in our study. The anti-methanogenic mechanisms of tomato fruit ingredients remain yet unknown [7], but either the extraction of the bioactive compounds during the industrial processing of tomato or their destruction by the heat applied in the process might help to explain the lack of anti-methanogenic properties of TP. The reduction of the CH_4_/total VFA ratio observed in TP-diets after 8 h of incubation could be due to the fermentation of soluble and rapidly degraded fractions, which also contributed to greater propionate proportions.

## 5. Conclusions

Tomato pomace is a high-moisture, fibrous by-product, but its dry matter contains a relatively high content of CP and EE and it is rapidly fermented in the rumen. Both the chemical composition and in vitro rumen fermentation of TP samples obtained from two processing plants at different times over the tomato campaign presented little variability. Crude protein of TP had low degradability in the rumen (average 510 g/kg) indicating a high bypass fraction, but the in vitro intestinal digestibility of the undegraded CP was low (average 459 g/kg). In vitro results indicated that dried TP could be included up to 180 g/kg in a high-concentrate diet without negative effects on rumen fermentation. However, TP did not show the anti-methanogenic properties previously reported for fresh tomato wastes, which was attributed to chemical changes produced during tomato processing.

## Figures and Tables

**Table 1:** Ingredients and chemical composition of experimental diets containing increased amounts of tomato pomace (TP) used in Experiment 2.

Item	Control	TP6	TP12	TP18
Ingredient (g/kg fresh matter)				
Barley	315	315	315	315
Corn	252	275	284	285
Wheat	130	130	130	130
Barley Straw	120	85.0	35.0	20.0
Soybean meal 46%	110	96.0	66.0	60.0
Wheat bran	48.1	20.0	40.0	-
Tomato pomace	-	60.0	120	180
Calcium soap	15.0	15.0	-	-
Calcium carbonate	5.0	5.0	5.0	5.0
Mineral/vitamine premix	5.0	5.0	5.0	5.0
Chemical composition ^1^				
Dry matter	903	903	902	905
Organic matter	945	957	969	970
Crude protein	145	145	145	145
Neutral detergent fiber	245	242	245	250
Acid detergent fiber	100	107	111	126
Ether extract	44.8	46.2	46.3	52.2
Non structural carbohydrates	510	524	533	523

^1^ Calculated from analyzed composition of individual feed ingredients. All chemical fractions are expressed as g/kg dry matter, except dry matter (g/kg fresh matter). Non-structural carbohydrates were calculated as the difference between organic matter and the sum of crude protein, neutral detergent fiber and ether extract.

**Table 2:** Chemical composition (g/kg dry matter (DM) unless stated otherwise) of tomato pomace samples taken from two processing plants at weekly intervals during the tomato harvesting campaign.

Item	Sampling Time (Weeks)						Average	SEM ^1^	*p*-Value
	1	2	3	4	5	6			
DM (g/kg)	260	277	244	276	231	291	263	36.2	0.841
Organic matter	961	965	957	962	963	970	963	3.5	0.350
Crude protein	173	150	160	158	159	161	160	8.4	0.623
Acid detergent insoluble N (ADIN)	2.22	1.58	2.07	2.08	2.12	2.24	2.05	0.146	0.126
Neutral detergent fiber	541	574	565	589	576	584	572	9.5	0.096
Acid detergent fiber	408	442	434	466	442	442	439	9.4	0.063
Acid detergent lignin	217 ^a^	244 ^ab^	229 ^ab^	259 ^b^	246 ^ab^	270 ^b^	244	7.2	0.017
Ether extract	107 ^c^	101 ^bc^	76.5 ^ab^	69.7 ^a^	81.9 ^ab^	84.4 ^ab^	82.7	5.36	0.015
Total sugars	123	124	134	108	113	127	122	15.4	0.856
Total soluble polyphenols	4.05	4.36	3.42	3.40	3.05	2.55	3.47	0.408	0.137
Potential DM degradability	595	574	614	583	595	519	580	27.0	0.323

^abc^ Within each variable, mean values with different superscripts differ (*p* < 0.05). ^1^ SEM = standard error of the mean.

**Table 3:** Gas production kinetics and fermentation parameters of tomato pomace samples taken from two processing plants at weekly intervals during the tomato harvesting campaign and samples.

Item	Sampling Time (Weeks)						Average	SEM ^1^	*p*-Value
	1	2	3	4	5	6			
Gas production parameters ^2^									
PGP (mL/g dry matter (DM))	199 ^a^	201 ^ab^	226 ^b^	211 ^ab^	210 ^ab^	187 ^a^	206	5.9	0.001
c (%/h)	7.28	7.17	6.97	7.04	7.17	6.88	7.09	0.181	0.316
lag (h)	2.33	2.63	2.33	2.50	2.42	2.48	2.45	0.130	0.064
AGPR (mL/h)	8.51 ^ab^	8.32 ^ab^	9.39 ^b^	8.81 ^b^	8.73 ^b^	7.48 ^a^	8.54	0.262	0.001
DMED (g/kg)	324 ^c^	305 ^bc^	332 ^c^	313 ^b^	320 ^c^	276 ^a^	312	7.3	<0.001
Fermentation parameters ^3^									
pH	6.81 ^ab^	6.80 ^ab^	6.76 ^b^	6.75 ^b^	6.79 ^ab^	6.83 ^a^	6.79	0.015	0.004
Total volatile fatty acids (VFA; mmol/g DM)	6.21 ^ab^	6.34 ^b^	6.52 ^b^	6.37 ^b^	6.41 ^b^	5.78 ^a^	6.27	0.25	0.004
Molar proportions (mol/100 mol)									
Acetate	66.8 ^ab^	65.9 ^a^	66.3 ^ab^	66.3 ^ab^	67.0 ^b^	66.8 ^ab^	66.5	0.23	0.007
Propionate	23.0 ^ab^	23.5 ^ab^	23.6 ^b^	23.5 ^ab^	23.0 ^ab^	22.7 ^a^	23.2	0.21	0.014
Butyrate	7.17	7.41	7.44	7.36	7.23	7.37	7.33	0.068	0.043
Minor VFA	2.98 ^ab^	3.14 ^b^	2.64 ^a^	2.82 ^ab^	2.78 ^ab^	3.10 ^b^	2.91	0.105	0.011
Acetate/propionate (mol/mol)	2.91 ^ab^	2.80 ^a^	2.81 ^ab^	2.83 ^ab^	2.93 ^ab^	2.95 ^b^	2.87	0.035	0.009
NH_3_ (mg/L)	173	158	155	156	159	155	159	8.2	0.608
CH_4_ (mL/g DM)	65.2 ^ab^	63.8 ^b^	75.0 ^b^	76.3 ^a^	65.0 ^ab^	61.8 ^a^	67.9	4.02	0.060
CH_4_/VFA (mL/mmol)	10.1	9.33	10.3	10.6	9.91	10.6	10.1	0.346	0.112

^ab^ Within each parameter, mean values with different superscripts differ (*p* < 0.05). ^1^ SEM: Standard error of the mean. ^2^ PGP: Asymptotic gas production (mL/g DM); c: Fractional rate of gas production (%/h); lag: Time before starting gas production; AGPR: Average gas production rate; DMED: Dry matter effective degradability calculated for a rumen particulate outflow of 4%/h. **^3^** Minor VFA: Calculated as the sum of isobutyrate, isovalerate and valerate.

**Table 4:** In situ rumen degradability of dry matter (DM) and crude protein (CP) after 16 h of incubation, in vitro intestinal digestibility of CP (IDCP), and total CP digestibility of tomato pomace samples taken from two processing plants at weekly intervals during the tomato harvesting campaign.

Item	Sampling Time (Weeks)						Average	SEM ^1^	*p*-Value
	1	2	3	4	5	6			
In situ degradability of DM (g/kg)	540 ^b^	523 ^ab^	530 ^ab^	488 ^a^	492 ^a^	489 ^a^	510	11.0	0.005
In situ degradability of CP (g/kg)	563	515	492	511	502	478	510	26.6	0.344
IDCP (g/kg)	450	430	467	466	475	467	459	19.0	0.593
Total CP digestibility (g/kg)	761	730	726	739	741	722	737	10.7	0.190

^ab^ Within each parameter, mean values with different superscripts differ (*p* < 0.05). ^1^ SEM: Standard error of the mean.

**Table 5:** Correlation matrix (Pearson coefficient and *p*-values in brackets; *n* = 12) of chemical composition of tomato pomace samples with gas production parameters, dry matter effective degradability (DMED), fermentation parameters measured in 24-h in vitro incubations, in situ rumen degradability of dry matter (DM) and crude protein (CP) after 16 h of incubation, and in vitro intestinal digestibility of CP (only *p* < 0.10 values are shown) ^1^.

Item	OM ^2^	NDF	ADF	Lignin	Lignin/NDF	N	EE	TSP
Gas production parameters ^3^								
PGP	0.860 (<0.001)		0.552 (0.063)		−0.590 (0.044)			
AGPR	−0.892 (<0.001)			−0.646 (0.023)				
DMED	−0.684 (0.014)			−0.770 (0.003)	−0.741 (0.006)			
Fermentation parameters								
Total volatile fatty acid (VFA) production	−0.857 (<0.001)		0.549 (0.064)		−0.613 (0.034)			
CH_4_	−0.789 (0.002)		0.617 (0.033)					
In situ degradability and in vitro intestinal digestibility								
In situ rumen DM degradability		−0.838 (0.001)	−0.726 (0.008)	−0.699 (0.011)			0.600 (0.039)	0.559 (0.059)
In situ rumen CP degradability			−0.724 (0.008)			0.796 (0.002)		
In vitro intestinal digestibility of CP			0.568 (0.054)				−0.511 (0.090)	−0.744 (0.006)
Total digestibility of CP			−0.516 (0.086)			0.790 (0.002)		

^1^ Total sugars and acid detergent insoluble N (ADIN) were not correlated to any of the analyzed parameters. ^2^ OM: Organic matter; NDF: Neutral detergent fiber; ADF: Acid detergent fiber; EE: Ether extract; TSP: Total soluble polyphenols. ^3^ PGP: Potential gas production (mL/g dry matter); AGPR: Average gas production rate until it has reached half of PGP (mL/h); DMED: Calculated for a rumen particulate outflow of 4% per h (%).

**Table 6:** Gas production parameters and fermentation parameters after 8 and 24 h of in vitro fermentation of diets containing increased amounts of tomato pomace incubated in batch cultures of mixed rumen microorganisms ^1^.

Item and Incubation Time	Control	TP6	TP12	TP18	SEM ^1^	*p*-Value	
						Lineal	Quadratic
Gas production parameters ^2^							
PGP (mL/g dry matter (DM))	336	334	326	334	3.67	0.453	0.229
c (%/h)	4.17 ^a^	4.62 ^b^	4.73 ^b^	4.58 ^b^	0.121	0.016	0.015
lag (h)	1.63	1.42	2.03	1.90	0.30	0.325	0.894
AGPR mL/h	9.16	10.1	9.74	9.76	0.37	0.416	0.236
DMED (%)	33.4	35.4	34.1	33.3	0.73	0.983	0.175
Fermentation parameters at 8 h incubation ^3^							
Total VFA (mmol/g DM)	3.47	3.44	3.52	3.62	0.075	0.161	0.430
Molar proportions (mol/100 mol)							
Acetate (Ac)	57.7 ^a^	58.3 ^b^	58.1 ^b^	58.2 ^b^	0.12	0.059	0.038
Propionate (Pr)	23.9 ^a^	23.9 ^a^	24.4 ^b^	24.4 ^b^	0.13	0.007	0.971
Butyrate	15.0 ^b^	14.6 ^a^	14.4 ^a^	14.4 ^a^	0.09	0.009	0.053
Minor VFA	3.37 ^c^	3.18 ^b^	3.08 ^a^	3.04 ^a^	0.043	0.002	0.095
Acetate/propionate (mol/mol)	2.46 ^ac^	2.48 ^ac^	2.42 ^ab^	2.42 ^a^	0.022	0.039	0.405
NH_3_-N (mg/L)	185 ^b^	182 ^ab^	181 ^ab^	178 ^a^	2.1	0.049	0.815
CH_4_ (mL/g DM)	18.5	18.0	18.0	18.4	0.36	0.901	0.296
CH_4_/VFA (mL/mmol)	5.34 ^b^	5.24 ^ab^	5.16 ^ab^	5.09 ^a^	0.082	0.047	0.836
FOM (mg/g)	332	328	334	345	7.2	0.178	0.325
Fermentation parameters at 24 h incubation ^3^							
pH	6.71 ^c^	6.71 ^c^	6.67 ^b^	6.63 ^a^	0.011	0.003	0.222
Total VFA (mmol/g DM)	6.82 ^a^	6.84 ^a^	6.91 ^ab^	6.99 ^b^	0.050	0.029	0.610
Molar proportions (mol/100 mol)							
Acetate (Ac)	58.6	58.8	58.4	58.5	0.07	0.150	0.569
Propionate (Pr)	19.4 ^a^	19.6 ^a^	20.1 ^b^	20.1 ^b^	0.14	0.002	0.594
Butyrate	17.5	17.4	17.5	17.5	0.10	0.705	0.576
Minor VFA	4.40 ^b^	4.14 ^b^	4.10 ^b^	3.96 ^a^	0.071	0.002	0.421
Ac/Pr (mol/mol)	3.08 ^b^	3.05 ^b^	2.96 ^a^	2.96 ^a^	0.022	0.007	0.465
NH_3_-N (mg/L)	284 ^b^	270 ^b^	271 ^b^	269 ^a^	4.8	0.066	0.215
CH_4_ (mL/g dry matter)	43.1	41.8	41.0	42.4	1.38	0.658	0.371
CH_4_/VFA (mL/mmol)	6.33	6.11	5.93	6.05	0.213	0.312	0.450
FOM (mg/g)	660 ^a^	661 ^a^	668 ^ab^	680 ^b^	4.3	0.007	0.293

^abc^ Within each parameter, mean values with different superscripts differ (*p* < 0.05). ^1^ Control, TP6, TP12, and TP18 diets contained 0, 60, 120, and 180 g of tomato pomace per kg, respectively (fresh matter basis). ^2^ PGP: Asymptotic gas production; c: Fractional rate of gas production; lag: Time before starting gas production; AGPR: Average gas production rate; DMED: Dry matter effective degradability calculated for a rumen particulate outflow of 4%/h. ^3^ VFA: Volatile fatty acids; minor VFA were calculated as the sum of isobutyrate, isovalerate, and valerate; FOM: Fermented organic matter calculated from acetate, propionate, and butyrate production as described by Demeyer [25].
